# Seasonal malaria chemoprevention in a context of high presumed sulfadoxine-pyrimethamine resistance: malaria morbidity and molecular drug resistance profiles in South Sudan

**DOI:** 10.1186/s12936-023-04740-x

**Published:** 2023-11-10

**Authors:** Irene Molina-de la Fuente, María José Sagrado Benito, Estrella Lasry, Janet Ousley, Luz García, Vicenta González, Harriet Akello Pasquale, Ahmed Julla, Piex Uwiragiye, Abdirashid M. Abdi, Buai Tut Chol, Bakri Abubakr, Agustín Benito, Cristian Casademont, Pedro Berzosa, Carolina Nanclares

**Affiliations:** 1 Alcala University, Madrid, Spain; 2 Institute of Health Carlos III, Madrid, Spain; 3 Médecins Sans Frontières, Carrer de Zamora, 54, 08005 Barcelona, Spain; 4 Médecins Sans Frontières, New York, NY USA; 5 National Malaria Control Programme, Ministry of Health, Juba, South Sudan; 6 Médecins Sans Frontières, Juba, South Sudan; 7 Médecins Sans Frontières, Nairobi, Kenya; 8 Centro de Investigación Biomedica en Red de Enfermedades Infecciosas (CIBERINFEC), Madrid, Spain

**Keywords:** SMC, Sulfadoxine-pyrimethamine plus amodiaquine, Haplotype mutations, South Sudan

## Abstract

**Background:**

Seasonal malaria chemoprevention (SMC) using sulfadoxine-pyrimethamine plus amodiaquine (SP-AQ), is a community-based malaria preventive strategy commonly used in the Sahel region of sub-Saharan Africa. However, to date it has not been implemented in East Africa due to high SP resistance levels. This paper is a report on the implementation of SMC outside of the Sahel in an environment with a high level of presumed SP-resistance: five cycles of SMC using SPAQ were administered to children 3–59 months during a period of high malaria transmission (July–December 2019) in 21 villages in South Sudan.

**Methods:**

A population-based SMC coverage survey was combined with a longitudinal time series analysis of health facility and community health data measured after each SMC cycle. SMC campaign effectiveness was assessed by Poisson model. SPAQ molecular resistance markers were additionally analysed from dried blood spots from malaria confirmed patients.

**Results:**

Incidence of uncomplicated malaria was reduced from 6.6 per 100 to an average of 3.2 per 100 after SMC administration (mean reduction: 53%) and incidence of severe malaria showed a reduction from 21 per 10,000 before SMC campaign to a mean of 3.3 per 10,000 after each cycle (mean reduction: 84%) in the target group when compared to before the SMC campaign. The most prevalent molecular haplotype associated with SP resistance was the IRNGE haplotype (quintuple mutant, with 51I/59R/108N mutation in *pfdhfr* + 437G/540E in *pfdhps*). In contrast, there was a low frequency of AQ resistance markers and haplotypes resistant to both drugs combined (< 2%).

**Conclusions:**

The SMC campaign was effective and could be used as an additional preventive tool in seasonal malaria settings outside of the Sahel, especially in areas where access to health care is unstable. Malaria case load reduction was observed despite the high level of resistance to SP.

**Supplementary Information:**

The online version contains supplementary material available at 10.1186/s12936-023-04740-x.

## Background

With over 2 million malaria cases and more than 4000 deaths in 2019, South Sudan has seen a steady increase in national malaria incidence and mortality trends, similarly to other countries in sub-Saharan Africa where progress against malaria has stalled since 2015. Malaria is a leading cause of morbidity and mortality, transmission is high throughout the year, seasonal peaks are impactful and differ by region of the country [1, 2].

Seasonal malaria chemoprevention (SMC) is a highly effective community-based intervention recommended by the World Health Organization (WHO) to prevent infections in areas with high malaria burdens and highly seasonal transmission [3]. It is administered through intermittent monthly administration of a full therapeutic course of anti-malarials, including a single dose of sulfadoxine–pyrimethamine (SP) plus three daily doses of amodiaquine (AQ), to children during the rainy season [3]. SPAQ provides protection from malaria infections for a period of approximately 4 weeks [4].

Since 2012, SMC has been implemented in 13 countries in the Sahel sub-region [5], the semi-arid expanse in West and Central Africa. Despite its demonstrated efficacy [6], it has never been systematically implemented outside the Sahel. Though interest in the strategy is growing and some recent pilot interventions have been attempted [7, 8], it is feared that widespread resistance to the components of SPAQ in East and South Africa will impact the intervention's effectiveness despite the fact that SMC and other chemo-preventive strategies’ efficacy in these contexts remains unclear[9, 10]. Most resistance surveillance studies are based on the genotyping of molecular resistance markers, but correlation between single nucleotide polymorphism (SNPs) and clinical and preventive outcomes is uncertain [9, 11]. Moreover, information on resistance to anti-malarials is scarce in South Sudan. SP resistance is associated with cumulative mutations in the *pfdhfr* and *pfdhps* genes, defining haplotypes with different levels of resistance [11], markers that have been shown to be highly prevalent and under selective pressure in different countries of East and Central Africa, such as Sudan and Democratic Republic of Congo (DRC) [9]. Resistance to AQ has also been related to polymorphism in the *Plasmodium falciparum* multidrug resistance gene (*pfmdr1*) and the chloroquine resistance transporter gene (*pfcrt*) [12].

As a result, an evaluation of these markers was carried out to provide a baseline study of SMC efficacy and to monitor the impact of SMC on resistance profiles in a non-Sahelian context [9, 11]. This study aims to describe the feasibility and effectiveness of an SMC campaign in a protracted humanitarian crisis in East Africa, in an area of presumed high-SP resistance. Additionally, it characterizes anti-malarial resistance genetic profiles after SMC in South Sudan.

## Methods

### Study site

The SMC campaign was conducted in 21 villages in rural areas of Yambio County, Western Equatoria State—a malaria endemic area with year-round transmission and a seasonal peak during the rainy period (July–December) [13].

The campaign was a collaboration between Médecins Sans Frontières (MSF), present in South Sudan since 1983, and the National Malaria Control Programme (NMCP) of the South Sudan Ministry of Health (MoH).

### Campaign design

Using information from the local census and data collected by MSF teams whilst implementing community activities, the population for the 21 villages was estimated at 109,435 people (Additional file 1: Table S1).

### Patient and public involvement

This time-series study using primary data was designed and conducted without patient and public involvement.

### SMC distribution

A total of 15,000 children (3–59 months of age) in 21 villages were targeted by the SMC campaign. Exclusion criteria followed WHO’s SMC protocol [14]. Prior to SPAQ distribution, community engagement and communication occurred. Given the duration of the malaria transmission season in Yambio, the campaign was extended to include 5 monthly SMC cycles (July 22nd to December 12th, 2019) (Table 1). Door-to-door distribution was not implemented due to security constraints. Instead, SPAQ was delivered using fixed distribution points such as health facilities, schools, churches, markets and previously established malaria community case management sites, located in areas with high population density, and within a 5 km radius of targeted villages.

**Table 1:** Administrative coverage and estimated coverage by household survey per cycle

	1st Cycle	2nd Cycle	3rd Cycle	4th Cycle	5th Cycle
Administrative coverage per cycle					
Target population	15,025	15,025	15,025	15,025	15,025
Children who received SMC	5862	7776	9510	11,290	13,689
Children who were excluded from SMC	2352	1197	515	393	415
Administrative coverage	39.0%	51.8%	63.3%	75.1%	91.1%
Dates	22 July–3 August	19–31 August	16–26 September	31 October–12 November^*^	2–12 December
Epi—weeks	30–33	34–37	38–42	43–48	49–52
Estimated coverage by household survey per cycle confirmed either by child record card or verbal report of participation					
Children who received the SMC N = 2674^	1634	1806	2005	2169	2341
Estimated coverage (%) (95% CI)	61.1% 59.23–62.9)	67.5% (65.73–69.3)	74.9% (73.29–76.6)	81.1% (79.58–82.6)	87.6% (86.24–88.8)

^*^4th cycle was postponed due to a simultaneous measles vaccination campaign in the area

^14 children were excluded from this analysis because they had missing data, 2674 was the total number of children included in the analysis

The SPAQ dosage was 250/12.5 mg SP and 75 mg AQ for children 3–11 months, while children 12–59 months received a double dose. SP single doses and the first dose of AQ were administered under directly observed treatment (DOT). The two remaining doses of AQ were provided by the caregiver at home on days 2 and 3. Adverse events were monitored and registered in national pharmacovigilance forms and actively sought in the SMC coverage survey.

### Malaria morbidity surveillance

Malaria incidence was estimated using cases of confirmed malaria reported by community health workers responsible for community case management in 12 of the 21 SMC sites from epidemiological week 10–52 (2019). Active case detection data was also collected during SMC distribution in all sites. Data on severe malaria cases was obtained from Yambio State Hospital (Yambio town) and from the NMCP Yambio malaria focal point.

### Coverage survey

A coverage survey was conducted at the household level 6 weeks after the end of the last SMC cycle. The sample size was calculated using a 60% expected coverage rate with an alpha error of 0.05, 5% precision, and a design effect of 2. This produced a total sample of 803 children aged from 3 to 59 months of age and a total of 1022 surveyed households from 28 clusters of 34 households each.

A two-stage cluster sampling methodology adapted the standardized WHO recommended method. Cluster allocation was systematically sampled, and the allocation probability was proportional to the respective population size of each sector/field.

### Molecular markers of resistance to anti-malarial drugs

Sample size was calculated using an expected prevalence of SP and AQ resistance of 50%, plus 20% for contingency. A total of 549 pLDH-based-RDT (CareStart™)-positive samples plus 45 RDT-negative samples were included.

Blood was collected from children with symptomatic malaria infections in 8 of 12 sites with CHW, selected considering logistic and security constrains, and at Yambio State Hospital from January to February 2020, at the end of the high malaria transmission season. Written informed consent was obtained from all participants. Dried blood spots (DBS) were sent and analysed in the Spanish National Centre of Tropical Medicine at the Carlos III Health Institute.

DNA was extracted from DBS on filter paper using the Chelex–Saponin method [15]. Malaria diagnoses were confirmed with malaria nested multiplex polymerase chain reaction (PCR) [16]. Samples with confirmed *Plasmodium falciparum* malaria were selected to examine SNPs.

Genotypes associated with SP resistance were studied through the mutations in the codons 51, 59, 108 and 164 in *pfdhfr* and 437, 540, and 581 in *pfdhps*. Molecular markers related with AQ resistance and other anti-malarial drugs were analysed by detecting mutations in positions 86 and 1246 in *pfmdr1* and 76 in *pfcrt* [17].

Mutation screening was performed according to previously published protocol with minor modifications [15] including lower temperature for denaturing (92ºC) and for extension (65ºC). Gene fragments were amplified by nested-PCR and then digested with different restriction enzymes (New England BiolabsR Inc.) to analyse restriction-fragment length polymorphisms (RFLPs) (Additional file 1: Table S2). This technique is based on the detection of fragments of different lengths after digestion of the samples with restriction enzymes depending on the presence of mutations (Additional file 1: Fig. S1). Haplotypes related with resistance were defined by combination of mutations as described by Naidoo et al*.* [18]: Quadruple mutants were considered partially resistant (IRNG); quintuple mutants were considered fully resistant (IRNGE); sextuple mutants were considered super resistant (IRNGEG). The haplotypes related with resistance to AQ and other anti-malarial drugs comprised one double mutation in a single gene: 86Y/1246Y *pfmdr1* and a combination of two single mutations in different genes: 86Y *pfmdr1* + 76 T *pfcrt*.

### Data analysis

A Poisson regression model was used to measure SMC's influence on the incidence of uncomplicated and severe malaria and assess differences in malaria morbidity trends by SMC cycle, adjusted by age group. Six time periods were established corresponding to each SMC cycle (Table 1), with each period comprising 4 weeks (except for period 4, which was 6 weeks long when the SMC cycle was delayed by a concurrent measles vaccination campaign in the area). Period 0 was the 4-week period prior to the start of the first SMC cycle (weeks 26–29) and was considered a baseline measure. Each 4-week period began with the SPAQ distribution. Incidence rate ratios were calculated comparing malaria incidence for each SMC cycle with the 4-week period prior to the start of the intervention (Period 0), which was also the beginning of peak malaria transmission. Comparisons were also made between the SMC target group (children < 5 years old) and the 5–14 year old age group. P-values of < 0.05 were considered statistically significant.

The frequency of SNP mutations and haplotypes were calculated as the number of haplotypes or mutations over the total *P. falciparum* cases in the study. To report the frequency of haplotypes, each haplotype has been considered independently, namely quintuple mutants have been included in the triple, quadruple and quintuple mutant frequencies. All data were managed in Microsoft Excel before being exported to R v4.0 for analysis.

## Results

A total of 48,127 doses of SPAQ were administered to children aged 3–59 months over 5 monthly cycles.

### SMC campaign implementation

The administrative coverage of SMC, estimated from the number of doses administered in the target population, progressively increased throughout the intervention, reaching 91.1% during the last cycle (Table 1). No serious adverse events were recorded during the 30-min post-administration observation period at any SMC site.

### SMC household coverage survey

A total of 2688 children aged 3–59 months were surveyed during the intervention, including 952 households distributed in 28 clusters (Additional file 1: Table S3). SMC coverage was verified by inspecting participants’ SMC child record card and by verbal confirmation of participation. A gradual increase in coverage was observed across consecutive SMC periods (Table 1), with 53.3% of children receiving at least four cycles. A notable 99% of people reported that they completed the full SMC treatment course (1 dose of SP and 3 doses of AQ) in each cycle. Among the < 1% who did not complete treatment, 69% responded that a caregiver forgot to administer the child’s medication on day 2 or 3.

The coverage survey collected information on adverse events, with 23.3% of surveyed children reporting having been unwell after receiving SMC. Among those reporting an adverse event, 46.1% experienced vomiting, 30.3% fever, 10.9% diarrhoea, 4.8% rash, 0.4% ‘yellow eyes’ (jaundice) and 7.5% ‘other.’

### Reduction in malaria morbidity

**Fig. 1:**
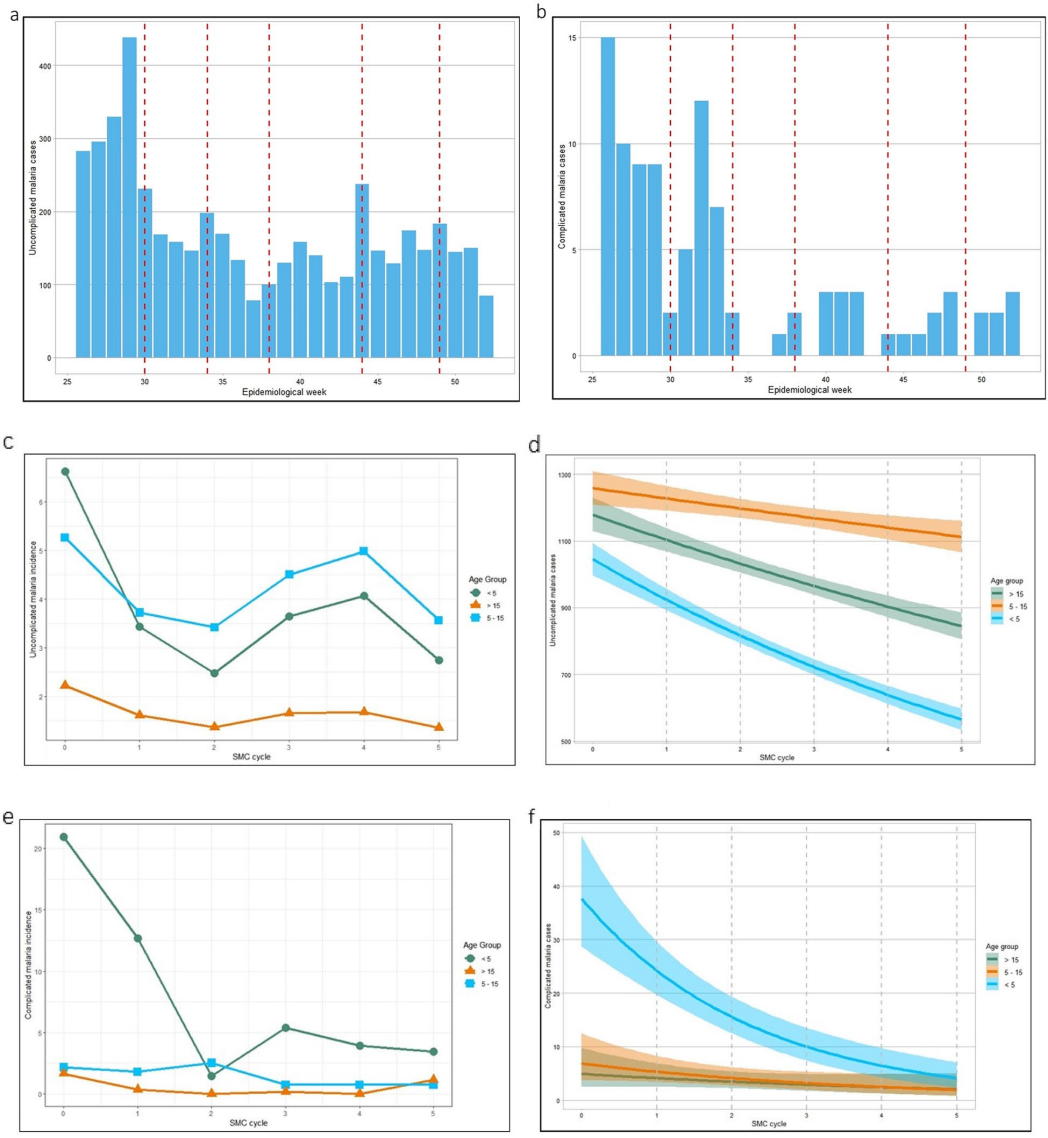
Impact of SMC campaign in malaria incidence indicators. **a** Weekly uncomplicated malaria incidence in children under five years. Malaria cases were represented as counts. Red lines represent the beginning of each SMC cycle. **b** Weekly severe malaria incidence in children under five. Malaria complicated cases were represented as counts. Red lines represent the beginning of each SMC cycle. **c** Uncomplicated malaria incidence per 100 people by age group. **d** Poisson model of uncomplicated malaria incidence by age group. **e** Severe malaria incidence per 10,000 people by age group. **f** Poisson model of severe malaria incidence by age group

The reduction of uncomplicated and severe malaria incidence compared to period 0 (before the SMC campaign) was observed in children < 5 years in all cycles (Fig. 1). Poisson regression analysis confirmed that SMC was associated with a significant decrease in incidence (p < 0.01). The highest reduction of uncomplicated malaria (63%) was seen during period 2 (Table 2). Additionally, while 5–15-year-olds (those not targeted by the SMC program) showed an overall decrease in malaria cases, the reduction was at least 20% higher in the SMC group (except during cycle 1, when the reduction was slightly lower: 19%). In children under five, the average reduction of severe malaria cases reported per cycle was 84% when compared to the period prior to the SMC intervention. The increase in malaria incidence observed in period 4 could be related to the longer duration of this cycle (6 weeks), surpassing the protection provided by SPAQ.

**Table 2:** Uncomplicated malaria incidence and percentual reduction in two different age groups in the intervention area (Incidence per100)

*SMC Cycle*	Weeks	Under five years of age				5–14 years of age			
		Incidence per 100	Estimate	IRR	Percent reduction	Incidence per 100	Estimate	IRR	Percent reduction
*0*	26–29	6.62	Ref	1		5.26	Ref	1	
*1*	30–33	3.43	− 0.65*	0.52	48%	3.72	− 0.43*	0.71	29%
*2*	34–37	2.47	− 0.98*	0.37	63%	3.41	− 0.60*	0.65	35%
*3*	38–43	3.64	− 0.59*	0.55	45%	4.50	− 0.33*	0.86	14%
*4*	44–48	4.06	− 0.48*	0.61	49%	4.98	− 0.25*	0.95	5%
*5*	49–52	2.74	− 0.88*	0.41	59%	3.56	− 0.56	0.68	32%

Weeks represents epidemiological weeks. I: Incidence of uncomplicated malaria cases per 100 people by SMC cycle, the denominator was calculated according to estimated percentage of each age group (18.74% under five, 25.52% 5–14 years), total population was estimated to be 109,435 people. Estimate according to Poisson model (malaria cases ~ SMC cycle standardized by age group). IRR: Incidence rate ratio. Percent reduction: percentage of reduction in malaria incidence compare to period 0. *: p-value < 0.001

### Molecular markers of resistance to anti-malarial drugs

A total of 601 samples from different areas in Yambio County were collected for molecular analysis. 532 samples confirmed as *P. falciparum* by PCR (519 RDT positive samples; 13 RDT negative samples) were analysed to assess their anti-malarial drug resistance genomic profile.

**Fig. 2:**
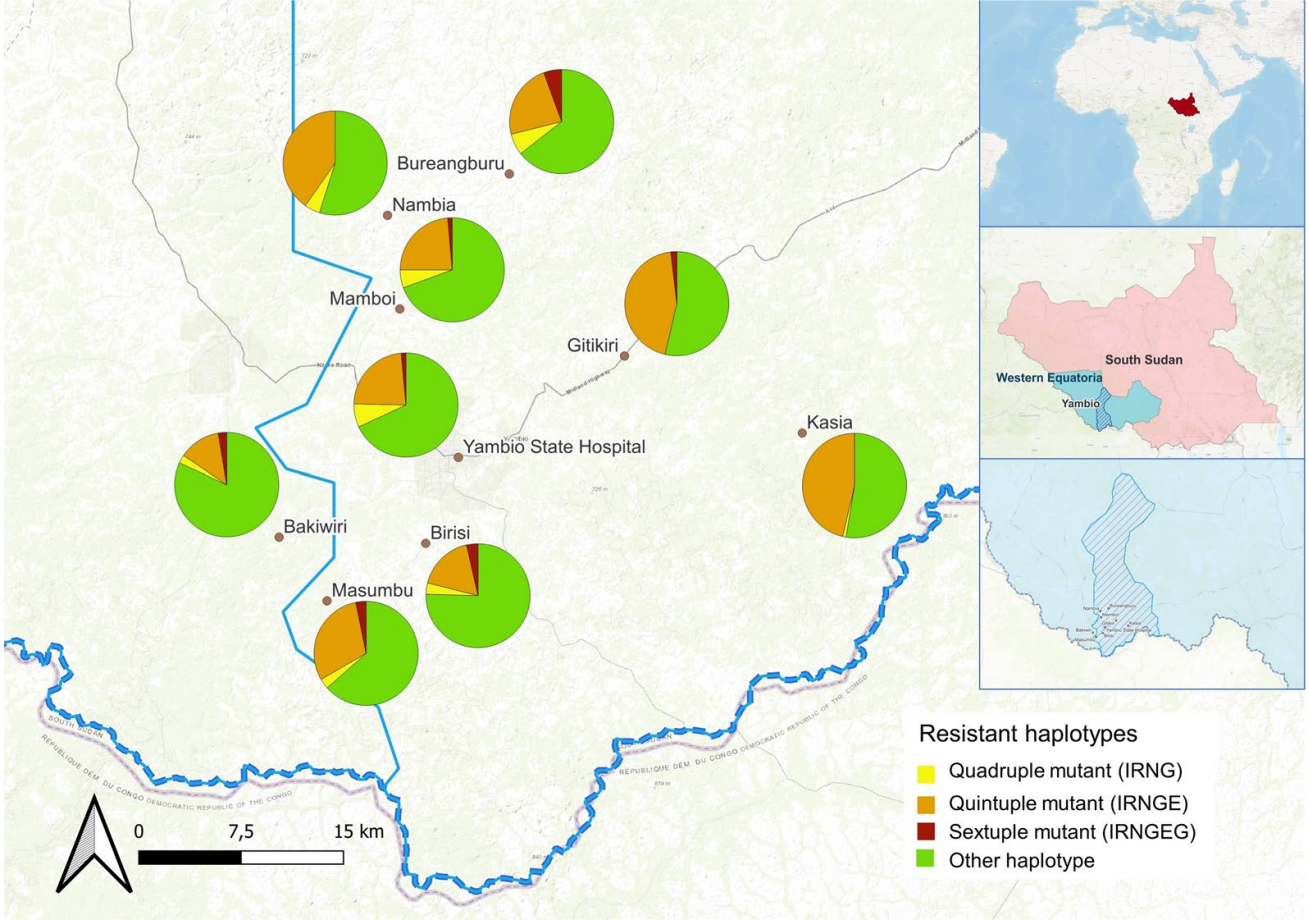
Map of the frequencies of resistant haplotypes to SP

Among molecular markers of SP resistance, mutations of the *pfdhfr* gene 51I (97.2%), 59R (84%) and 108N (97%) and of *pfdhps* 437G (81.7%) and 540E (86.5%) were frequently found. Mutations *pfdhfr* 164L (0.7%) and 581G (6.4%), both associated with high resistance, were less prevalent. Fully resistant haplotypes (IRNGE) were most commonly seen (62.4%) (Table 3) but slight differences between villages were observed (Fig. 2).

**Table 3:** Genotype profile of the different genes related with resistance to SP, AQ or the combination of both

No of mutations	Combination	Haplotype	Yambio (N = 532)	
			N (%)	95% CI
Genotype of genes related with resistance to SP				
*Pfdhfr*				
3	51I/59R/108N	IRN	446 (83.8%)	85–87
3	51I/108N/164L	INL	1 (0.2%)	0.0–1
4	51I/59R/108N/164L	IRNL	2 (0.4%)	0.1–1
*Pfdhfr* + *Pfdhps*				
4	51I/59R/108N/437G*	IRNG	367 (68.9%)	65–73
5	51I/59R/108N/437G/540E**	IRNGE	332 (62.4%)	58–66
6	51I/59R/108N/437G/540E/581G***	IRNGEG	24 (4.5%)	3–7
Genotype genes related with resistance to AQ and different anti-malarial drugs				
*Pfmdr1*				
1	86Y	Y	101 (18.9%)	16–22
1	1246Y	Y	51 (9.5%)	7–1
2	86Y/ 1246Y	YY	23 (4.3%)	3–6
*Pfcrt*				
1	76 T	T	113 (21.2%)	18–25
*Pfmdr1* + *Pfcrt*				
3	86Y/1246Y/76 T	YYT	16 (3%)	17–48
Combination of mutations related with SP and AQ resistance				
*Pfdhfr* + *pfdhps* + *pfmdr1*				
6	51I/59R/108N/437G/540E/86Y/	IRNGEY	8 (1.5%)	
7	51I/59R/108N/437G/540E/86Y/1246Y	IRNGEYY	7 (1.3%)	
7	51I/59R/108N/437G/540E/581G/86Y	IRNGEGY	1 (0.18%)	
*Pfdhfr* + *pfdhps* + *pfmdr1* + *pfcrt*				
8	51I/59R/108N/437G/540E/86Y/1246Y/76 T	IRNGEYYT	6 (1.12%)	

*: partially resistant

**: fully resistant

***: super resistant

Among molecular markers of resistance to other anti-malarial drugs, mutations 86Y and 1246Y of the *pfmdr1* gene, associated with decreased sensitivity to AQ and chloroquine, were found in 18.9% and 9.5% of samples respectively (Table 3). The combination of both mutations appeared less frequently (4.3%). Mutation 76 T of the *pfcrt* gene, associated with resistance to chloroquine, was found in 21% of samples.

The genotype combining the three mutations was detected in 3% of samples (Table 3). The prevalence of haplotypes combining mutations in all the genes related to resistance to SP and AQ were all under 2% (Table 3).

## Discussion

Though SMC has been well established as an effective malaria prevention approach, its administration to date has been in relatively homogenous environmental contexts across the Sahelian region of sub-Saharan Africa. By testing SMC’s utility and feasibility in a novel environment in South Sudan—conflict-affected, extremely remote and resource limited, with a high level of presumed SP resistance—the results of this study show that the intervention remains a feasible and effective tool in the effort to control malaria in a variety of highly endemic areas with seasonal transmission. Results of this study also provide more evidence supporting updated WHO guidance recommending SMC use in environments outside of the Sahel with highly seasonal malaria transmission and defining the number of cycles depending on patterns of malaria transmission [14]. One of the main threats to SMC implementation in Eastern Africa (compared to Central and Western regions) is the high level of reported SP resistance [19–21]. Yet, the interaction between resistance and SMC efficacy is still poorly understood. Notably, there is weak evidence for establishing specific molecular thresholds above which a particular chemoprevention strategy should not be implemented [8]. Moreover, the longer term use of SPAQ in SMC campaigns has not been shown to be related to an increase in SP resistance [10]. In South Sudan, SP molecular resistance is high, given that fully resistant haplotypes (IRNGE) were the most commonly found form. The high frequency of haplotypes related to SP resistance was similar to what has been described in neighbouring countries [11, 12, 22]. By pairing programmatic data with molecular outcomes, this study was able to provide insightful, actionable information to inform SMC and other chemoprevention activities [19, 23].

The study’s SMC campaign implementation overcame important barriers to scale-up and achieved satisfactory coverage despite the complex security context within which it occurred and the scattered population it targeted. Community engagement was an essential component to success: As community confidence in SMC grew, SMC acceptability also increased and was reflected by increasing coverage [24]. Population movements due to insecurity complicated accurate population estimates, yet the campaign remained feasible even in hard-to-reach areas with higher prevention needs due to limited health care and treatment options.

Although approximately a quarter of survey participants reported adverse events, this is likely to be overestimated due to miscommunication or the inclusion of events unrelated to SMC. Among the directly observed or reported adverse events seen, vomiting, fever and diarrhoea were most common, though no serious adverse events were reported, demonstrating SMC’s safety and its ability to be extended over 5 cycles without increasing the risk of serious side effects [25, 26]. Typically, SMC is only administered over 4 cycles (maximum) per year (though examples of 5-cycle SMC have increasingly been seen and are reflected in WHO guideline updates) [14, 27]. This 5-cycle campaign was necessary in the longer malaria transmission season and, though the approach risked potential ‘campaign fatigue’, it was observed that coverage was highest in the final cycles, remaining safe and acceptable to caregivers.

Although > 90% coverage was not achieved for all cycles, the campaign demonstrated a reduction in malaria morbidity comparable to other SMC interventions in Sahelian areas [28, 29]. More pronounced reductions in severe malaria (compared to uncomplicated cases) was also consistent with other research and could be explained by the fact that SMC prevents severe clinical disease but does not always protect children against infection [30]. Although a decrease in malaria morbidity was observed after each cycle in all age groups, the targeted children 3–59 months of age showed a significantly higher reduction, confirming SMC’s impact.

Quintuple mutants (IRNGE), of which there is limited evidence in East Africa, represented the majority of the parasite population in this study [20, 27, 31, 32]. Previously, the WHO recommendation was against using SP for intermittent preventive therapy for infants (IPTi) when mutation *pfdhps* 540E was higher than 50%. However, recently this guidance has changed, now asserting that molecular marker frequency does not predict chemoprevention effectiveness [14]. Supporting the newer position, this study found that SMC was effective despite very high levels of this mutation (87%).

In relation to neighbouring countries, the *pfdhps* 581G mutation was still low (6.4%) compared to the DRC (10%) and super resistant haplotypes (IRNGEG) were observed less frequently (4.5%) than in neighbouring Kenya (13.2%) [32]. However, this mutation is expected to gradually increase and become unequally distributed, similar to what has been seen in Uganda [21]. The *Pfdhfr* 164L mutation, highly associated with resistance to pyrimethamine, was detected at a low frequency (similar to Kenya) [32]. This mutation has not been detected in Sudan [20]. AQ resistance marker frequency was equal or lower to what has been reported in neighbouring countries and was not considered a concern for SMC efficacy [21, 22, 32]. The low frequency of haplotypes resistant to combined treatment (SPAQ) could explain the efficacy of SPAQ-based SMC campaigns.

Previous SMC experience has shown that monitoring the final 2 doses of AQ was impractical, and this South Sudanese experience supported that finding [33]. Despite uncertainty about adherence on days 2 and 3, a reduction in malaria morbidity was observed.

This study was limited by a lack of malaria surveillance data, access to retrospective malaria data, and the absence of a control group. Given that evidence of SMC efficacy is based on the comparison of data after each cycle to data before the campaign, it is acknowledged that other factors could have contributed to the decreasing trends observed. Furthermore, given the complexity of the setting, it was not feasible to follow up a cohort of children to assess SMC protection throughout the intervention period. Accurate population figures were difficult to obtain given the scattered and mobile communities targeted.

## Conclusion

This is the first study to assess a SMC intervention in East Africa and in a conflict affected area. It contributes insightful information on the operational implementation of SMC, including the feasibility of extending SMC campaigns over 5 cycles when necessary, as well as its effect on malaria morbidity and the resistance profiles of a unique SMC context with high SP resistance. In South Sudan, SPAQ-based SMC was feasible, safe, and contributed to a reduction in malaria morbidity. Security constraints and hesitancy from communities had an important impact on coverage in the first cycles that improved over time with community engagement. Despite the high frequency of resistance to SP, the reduction in malaria cases seen could indicate that that SPAQ may retain its protective effect.

The findings of this study align with updated WHO guidelines and provide evidence for SMC implementation in South Sudan and outside of the Sahel. Regular impact monitoring of health outcomes and SP/AQ resistance markers should occur.

### Supplementary Information

**Additional file 1.** Supplementary material 1A . Details of estimation of target population. **Table 1A**. Target population figures. Supplementary material 2A. Details of RFLPs for resistance markers. **Table 2A**. Primers and enzymes used for RFLPs study of resistance markers. Supplementary material 3. Summary of demographic information of the SMC survey. **Table 3A**. Demographic information of the surveyed population. **Table 3B**. Distribution of the number of HH clusters that were surveyed within the SMC sites. **Figure S1**. Electrophoresis gel after digestion with restriction enzymes.

## Data Availability

The study protocol and statistical analysis data that underlie the results reported in this article will be made available upon request. Proposals should be directed to the corresponding author. Requests will be reviewed and sharing of the data will follow the conditions required by all applicable laws and the possible prior signature of any necessary agreement, in accordance with the legal framework set forth by Médecins Sans Frontières (MSF) data sharing policy, which ensures that all security, legal, and ethical concerns are addressed.

## Abbreviations

AQ: Amodiaquine
CHW: Community health health workersworkers
DBS: Dried blood spots
DOT: Directly observed treatment
DRC: Democratic Republic of Congo
IPTi: Intermittent preventive therapy for infants
MoH: South Sudan Ministry of Health
MSF: Médecins Sans Frontières
NMCP: National Malaria Control Programme
PCR: Polymerase Chain Reaction
*pfcrt*: *Plasmodium falciparum* Chloroquine resistance transporter gene
*Pfdhfr*: *Plasmodium falciparum* Dihydrofolate reductase gene
*Pfdhps*: *Plasmodium falciparum* Dihydropteroate synthase gene
*Pfmdr*: *Plasmodium falciparum* Multidrug resistance gene
pLDH: *Plasmodium* Lactate dehydrogenase
RDT: Rapid diagnostic diagnostic testtest
RFLPs: Restriction-fragment length polymorphisms
SMC: Seasonal malaria chemoprevention
SNPs: Single nucleotide polymorphism
SP: Sulfadoxine-pyrimethamine
SPAQ: Sulfadoxine-pyrimethamine plus amodiaquine
WHO: World Health Organization
