# Supplementary material for: Seasonal malaria chemoprevention in a context of high presumed sulfadoxine-pyrimethamine resistance: malaria morbidity and molecular drug resistance profiles in South Sudan
Source: Malar J. 2023 Nov 10;22:345. doi: 10.1186/s12936-023-04740-x (PMC10637007; doi:10.1186/s12936-023-04740-x)
Supplement: Supplementary file 1 — Additional file 1. Supplementary material 1A . Details of estimation of target population. Table 1A. Target population figures. Supplementary material 2A. Details of RFLPs for resistance markers. Table 2A. Primers and enzymes used for RFLPs study of resistance markers. Supplementary material 3. Summary of demographic information of the SMC survey. Table 3A. Demographic information of the surveyed population. Table 3B. Distribution of the number of HH clusters that were surveyed within the SMC sites. Figure S1. Electrophoresis gel after digestion with restriction enzymes. [file 12936_2023_4740_MOESM1_ESM.pdf]

Supplementary material

Supplementary material 1A

Table 1A. Target population figures

Axis	Boma	Pop Estimates used for SMC Planning	Target population SMC (3m - 59m) 13.73%
**Li Rangu**	Nambia	1,884	259
	Momboi	1,701	234
	Li Rangu Centre	2,775	381
**Birisi**	Masumbu	6,942	953
	Nasoro	2,225	305
	Birisi Centre	4,164	572
	Bakiwiri	1,709	235
	Bureamburu	4,164	572
**Gangura**	Asanza	7,060	969
	Nangbimo	7,765	1,066
	Kidi	3,264	448
	Gangura Centre	7,539	1,035
	Nabiapai	5,905	811
**James Diko**	Bazungua	15,065	2,068
	Gitikiri	5,658	777
	Bodo	9,962	1,368
	Bazumburu	3,170	435
	Rimenze	6,300	865
	Kasia	5,358	736
	Nangere	4,626	635
	Ukuo	2,199	302
**TOTAL**		**109,435**	**15,025**

Supplementary material 2A

Table 2A.

gene	codon	PCR	primer, sequence (5´- 3´)	size	enzymes	aminoacids
Pfdhfr	**51/59**	**1st**	**FR519-A** 5'GCGCGCTAATAACTACACATTTA3'<br>**FR519-B** 5'CCCGGGCTCTTATATTTCAATTT3'			
		**2nd**	**FR51-D** 5'CTAGGAAATAAAGGAGTATTACCATGGAAATGGA3'<br>**FR59-D** 5'ATTTTTCATATTTTGATTCATTCACATATGTTGTAACTGTAC3'	**113bp**	**EcoRI** (co. 51)<br>**BsrGI** (co. 59)	Asn51Ile<br>Cys59Arg
	**108/164**	**1st**	**FR100-A** 5'GGGGGGCAGTTACAACATATGTGA3'<br>**FR100-B** 5'GGGGGCACATTCATATGTACTATTT3'			
		**2nd**	**FR108-D** 5'CTAATTCTAAAAAATTACAAAATGT3'<br>**FR164-D3** 5'TTTCTTTTCTAAAAATTCTTGATAAACAACGGAACCTCTTA3'	**254bp**	**Alu I** (co. 108)<br>**PsiI** (co. 164)	Ser108Asn<br>Ile164Leu
Pfdhps	**436/437**	**1st**	**PS400-A** 5'GGGGTATTAAATGTTAATTATGATTCT3'<br>**PS400-B** 5'GGGGTCACATTTAACAATTTTATT3'			
		**2nd**	**PS400-D1** 5'TGTTCAAAGAATGTTTGAAATGA3'<br>**PS400-D2** 5'CCATTCTTTTTGAAATAATTGTAAT3'	**148bp**	**Ava II** (co. 437)	Ala437Gly
	**540/581**	**1st**	**PS500-A** 5'GGGCCCAAACAAATTCTATAGTG3'<br>**PS500-B** 5'GGCCGGTGGATACTCATCATATA3'			
		**2nd**	**PS500-D1** 5'GCGCGCGTTCTAATGCATAAAAGAGG3'<br>**PS500-D2** 5'CCCGGGTAAGAGTTTAATAGATTGATCA*GC*TTTCTTC3'	**201bp**	**Fok I** (co. 540)<br>**Mwo I** (co. 581)	Lys540Glu<br>Ala581Gly
Pfmdr1	**86**	**1st**	**MDR-86A** 5'GCGCGCGTTGAACAAAAAGAGTACCGCTG3'<br>**MDR-86B** 5'GGGCCCTCGTACCAATTCCTGAACTCAC3'			
		**2nd**	**MDR-86D1** 5'TTTACCGTTTAAATGTTTACCTGC3'<br>**MDR-86D2** 5'CCATCTTGATAAAAAACACTTCTT3'	**291bp**	**Afl III** (codon 86)	Asn86Tyr
	**1246**	**1st**	**1246-A** 5'GGGGGATGACAAATTTTCAAGATTA3'<br>**1246-B** 5'GGGGGACTAACACGTTTAACATCTT3'			
		**2nd**	**1246-D1** 5'AATGTAAATGAATTTTCAAACC3'<br>**1246-D2** 5'CATCTTCTCTTCCAAATTTGATA3'	**203bp**	**Bgl II** (codon 1246)	Asp1246Tyr
Pfcrt	**76**	**1st**	**76-A** 5'GCGCGCGCATGGCTCACGTTTAGGTGGAG3<br>**76-B** 5'<br>GGGCCCGGCGGATGTTACAAAACTATAGTTACC3'			
		**2nd**	**76-D1** 5'TGTGCTCATGTGTTTAAACTT3'<br>**76-D2** 5'CAAAACTATAGTTACCAATTTTG'	**145bp**	**Apo I** (codon 76)	Lys76Thr

Supplementary material 3.

There were 7 original SMC sites (Nambia, Momboi, Li Rangu, Nasoro, Birisi, Nangere, and Ukuo) that were not surveyed in this study due to security and access issues or because they were not selecteded by the ENA program.

Table 3A. **Demographic information of the surveyed population**

**Demographic data**	
Total number of HHs sampled	952
Total number of persons 3-59 months	2,688
Average HH size	8.5 people
Sex –Female	1355 (50.9%)
Sex –Male	1333 (49.0%)
Number of children 3-11 months	246 (9.0%)
Number of children 12-59 months	2,442 (91.0%)

Table 3 B. Distribution of the number of HH clusters that were surveyed within the SMC sites.

**Axis**	**Village**	**Pop Estimates**	**HHs Surveyed (n, %)**
Birisi	Masumbu	6,942	68 (7.1%)
	Bakiwiri	1,709	34 (3.6%)
	Bureamburu	4,164	34 (3.6%)
Gangura	Asanza	7,060	68 (7.1%)
	Nangbimo	7,765	102 (10.7%)
	Kidi	3,264	34 (3.6%)
	Gangura Centre	7,539	68 (7.1%)
	Nabiapai	5,905	68 (7.1%)
James Diko	Bazungua	15,065	170 (17.9%)
	Gitikiri	5,658	68 (7.1%)
	Bodo	9,962	102 (10.7%)
	Bazumburu	3,170	34 (3.6%)
	Rimenze	6,300	68 (7.1%)
	Kasia	5,358	34 (3.6%)
**TOTAL**		**109,435**	**952**

Figure S1. Electrophoresis gel after digestion with restriction enzymes.

Fig 1Sa. Fragments of *pfdhfr* & *pfdhps* after digestion with restriction enzymes.

Pfdhfr **codons**

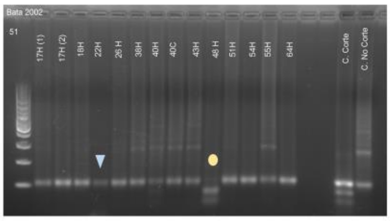

Codon 51: Cut 35 & 78 bp /No cut 113 bp

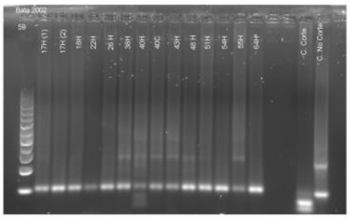

Codon 59: Cut 43 & 65 bp / No cut 113 bp

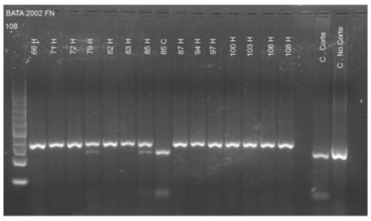

Codon 108: Cut 46 & 210 bp /No cut 254 bp

Pfdhps **codons**

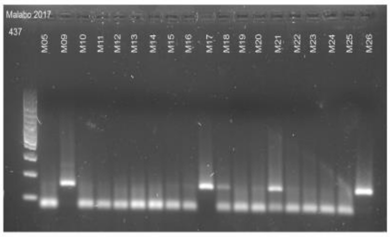

Codon 437: Cut 69, 79 bp /No cut 148 bp

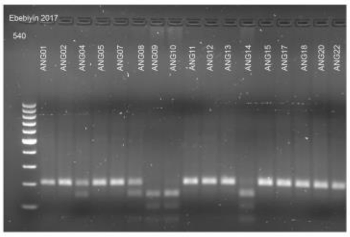

Codon 540: Cut 56, 145 bp / No cut 201 bp

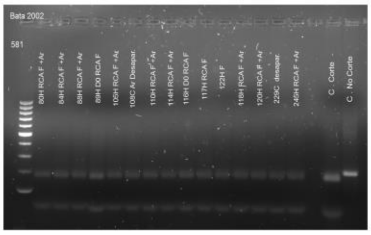

Codon 581: Cut 36 & 154 bp / No cut 201 bp

▼ No cut ● Cut

1Sa

Fig S1b. Fragments of *pfmdr1* & *pfcrt* after digestion with restriction enzymes.

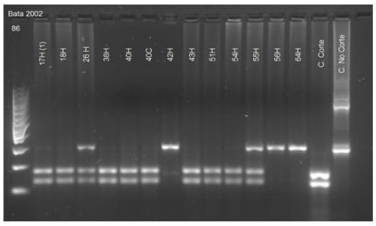

Codon *pfmdr1* 86: Cut 126 & 165 bp /No cut 295 bp

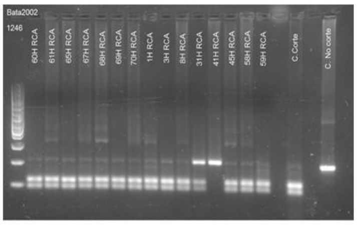

Codon *pfmdr1* 1246: Cut 90 & 113 bp / No cut 203 bp

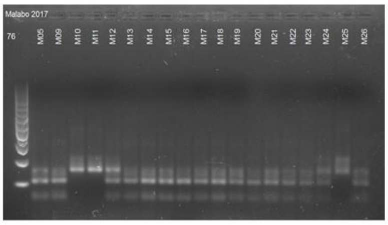

Codon *pfcrt* 76: Cut 46 & 99 bp / No cut 145 bp

1Sb
